# Prognostic Value of ATRX and p53 Status in High-Grade Glioma Patients in Morocco

**DOI:** 10.7759/cureus.56361

**Published:** 2024-03-18

**Authors:** Asmae Squalli Houssaini, Salma Lamrabet, Nadia Senhaji, Mohammed Sekal, Jean Paul Nshizirungu, Hajar Mahfoudi, Samira Elfakir, Mehdi Karkouri, Sanae Bennis

**Affiliations:** 1 Laboratory of Biomedical and Translational Research, Faculty of Medicine, Pharmacy, and Dental Medicine of Fez, Sidi Mohamed Ben Abdellah University, Fez, MAR; 2 Department of Biology, Faculty of Sciences, Moulay Ismail University, Meknes, MAR; 3 Laboratory of Epidemiology and Research in Health Sciences, Faculty of Medicine, Pharmacy, and Dental Medicine of Fez, Sidi Mohamed Ben Abdellah University, Fez, MAR; 4 Department of Biology, School of Science, College of Science and Technology, University of Rwanda, Kigali, RWA; 5 Laboratory of Epidemiology and Research in Health Sciences, Faculty of Medicine, Pharmacy and Dental Medicine of Fez, Sidi Mohamed Ben Abdellah University, Fez, MAR; 6 Department of Pathology, Ibn Rochd University Hospital Center, Casablanca, MAR; 7 Department of Pathology, Faculty of Medicine and Pharmacy, Hassan II University of Casablanca, Casablanca, MAR

**Keywords:** clinicopathological parameters, atrx, p53, idh, glioma

## Abstract

Introduction: Glioblastoma and astrocytoma, grade 4, are the most common and aggressive brain tumors. Several biomarkers, such as the isocitrate dehydrogenase mutation (IDH-1), alpha-thalassemia/mental retardation, and the X-linked mutation (ATRX), enable more accurate glioma classification and facilitate patient management. This study aimed to determine the prognostic value of clinical and molecular factors (IDH, TP53, and ATRX mutations). We also studied the relationship between these molecular markers and the overall survival (OS) of 126 patients with grade 4 glioblastoma/astrocytoma.

Methods: The immunohistochemical study was conducted using antibodies namely, IDH1, R132H, p53, and ATRX. Statistical tests were used to investigate factors that might influence overall survival using IBM SPSS Statistics, version 25.0 (IBM Corp., Armonk, NY).

Results: The median age at diagnosis was 51.5 years. Patients with a Karnofsky performance score (KPS) <70 presented less favorable survival outcomes compared to those with a KPS ≥70. The median OS for patients was found to be 11.17 months. Expression of IDH1 R132H was found in 13.5% of patients, p53 overexpression was identified in 55.6% of cases, and loss of ATRX expression was detected in 11.9%. The group of patients with IDH mutant/ATRX mutant/p53 wild-type had the best prognosis (OS = 27.393 months; p = 0.015). Our results were in line with previous studies.

Conclusion: The clinical value of IDH and ATRX mutations in prognostic assessment was confirmed (p ≤0.05). The overexpression of p53 had no significant impact on OS (p = 0.726). Therefore, p53 alone cannot predict survival in glioblastoma patients. Based on the results, these biomarkers may be a potential therapeutic target to prolong patient survival, hence the need for further investigations.

## Introduction

Astrocytoma and glioblastoma, grade 4, are the most common and aggressive brain tumors, accounting for 14.5% of all central nervous system tumors and 48.6% of malignant central nervous system tumors [1]. Their incidence increases with age and other factors such as ionizing radiation, inherited genetic disorders, and race or ethnicity [2].

Several alterations can contribute to gliomagenesis, including isocitrate dehydrogenase mutation (IDH-1), alpha-thalassemia/mental retardation, X-linked mutation (ATRX), and tumor protein p53 mutation (TP53). These mutations are of diagnostic and/or prognostic interest [2].

IDH-1 catalyzes the oxidative decarboxylation of isocitrate to alpha-ketoglutarate. In contrast, the mutant form of IDH-1 leads to the formation of 2-hydroxyglutarate, an oncometabolite involved in gliomagenesis by blocking dioxygenase functions in DNA and histone demethylation [3]. Several clinical trials investigating IDH-1 inhibitors among patients with gliomas are underway; however, their efficacy is not always approved [4].

The ATRX gene is located on Xq21.1 and encodes a protein involved in chromatin rearrangement [5]. The ATRX mutation is found in around 75% of grade 4 astrocytomas and rarely in IDH wild-type glioblastomas (only 3%) [6, 7]. In addition, patients with ATRX mutations have better overall survival (OS) than patients with wild-type ATRX [2,8,9].

The TP53 gene, located on 17p13.1, is a tumor suppressor involved in cell cycle and apoptosis. The mutant form of p53 contributes to tumor cell proliferation and facilitates the malignant transformation of astrocytic tumors. The presence of this mutation is often associated with the IDH-1 mutation (65%-90% of cases) [10-12]. In contrast, the TP53 mutation is present in only 30% of IDH wild-type glioblastomas [10]. Alteration of p53 expression leads to increased malignancy in glial cells by enhancing their proliferation, invasion, and resistance to chemotherapy [11]. In their study, Wang et al. reported that TP53 mutations are associated with a poor prognosis [12]. In astrocytomas, IDH-1 and TP53 mutations are also associated with the presence of the ATRX mutation [13]. Despite recent advances in treatment modalities, the prognosis remains dismal, with the OS ranging from 12 to 18 months [14].

This study aimed to examine the main clinicopathologic parameters and their association with patients' OS. We also determined the prognostic value of the IDHR132H mutation in association with p53 overexpression and ATRX loss in a series of 126 glioma patients.

## Materials and methods

Patients and tissue samples

The present retrospective study includes 126 patients with grade 4 glioma diagnosed and treated between 2016 and 2020 at the University Hospital of Casablanca, Casablanca, Morocco. Tissue samples were obtained by neurosurgical intervention, fixed in formalin, and embedded in paraffin (FFPE).

Demographic data, cerebral magnetic resonance imaging (MRI), location of the tumor, type, extent of resection, Karnofsky performance score (KPS), and treatment characteristics were collected.

Immunohistochemistry (IHC)

An immunohistochemical study was carried out using three antibodies: IDHR132H (clone H09, 1:20 dilution, ph9) from Dianova (BIOZOL, Berlin, Germany), p53 (clone DO-7, ready to use, pH9) from Dako (Agilent Technologies, Inc, Santa Clara, CA), and ATRX (clone BSB-108, ready to use, pH9) from Bio SB (Bio SB, Inc., Santa Barbara, CA). For each stain used, there was an appropriate control tissue to ensure that the stains worked correctly. 

Tissue sections of 4 µm thickness each were obtained using a microtome. The IHC staining followed the standard procedure of deparaffinization, antigen retrieval, and rehydration. These steps were performed using EnVision FLEX Target Retrieval Solution ((Agilent Technologies, Inc) at a high pH (x50). Endogenous peroxidase was blocked with EnVision FLEX Peroxidase Reagent (Agilent Technologies, Inc) for five minutes.

The slides were incubated with a primary antibody for 30 minutes, and then the visualization reagent (EnVision FLEX/HRP) containing dextran polymers, coupled with peroxidase molecules and biotinylated secondary antibody molecules, was applied for 20 minutes. 3,3′-diaminobenzidine tetrahydrochloride (DAB) was used for primary antibody detection. As a result, visible brown products were perceived. Later, hematoxylin was applied.

Slide examination

Slides were observed by two neuropathologists. Cases were considered mutant p53 if >10% of neoplastic cells showed nuclear staining. ATRX immunoreactivity was almost totally absent or completely retained; thus, cases with less than 10% of the stained nucleus were defined as ATRX loss.

Statistical analysis

The statistical study was carried out using IBM SPSS Statistics, version 25.0 (IBM Corp., Armonk, NY) (chi-squared (χ2) test and Fisher's exact test). The OS was estimated using the Kaplan-Meier method, and differences in survival within groups were examined using the log-rank test (Mantel-Cox). The influence of various parameters on survival outcomes was tested by univariate analysis. Results were considered statistically significant when p-values were less than 0.05.

## Results

Patient characteristics

The study included 126 cases of gliomas. The description of prognostic and clinicopathological factors is shown in Table 1.

**Table 1:** Demographic data, tumor characteristics, and treatment strategies of patients KPS: Karnofsky performance score The data have been represented as N

Clinicopathological characteristics	Age ≤40 years: 31/126 (24.6%)	Age >40 years: 95/126 (75.4%)
Female	14	37
Male	17	58
KPS at admission ≤70	3	26
KPS at admission >70	28	69
KPS at last evaluation ≤70	23	90
KPS at last evaluation >70	8	5
Frontal localization	17	39
Temporal localization	7	31
Parietal/Occipital localizations	7	25
Subtotal resection	13	51
Gross total resection	18	44
Postoperative radiotherapy	26	82
Postoperative chemotherapy	24	70
Death	27	94

**Figure 1:**
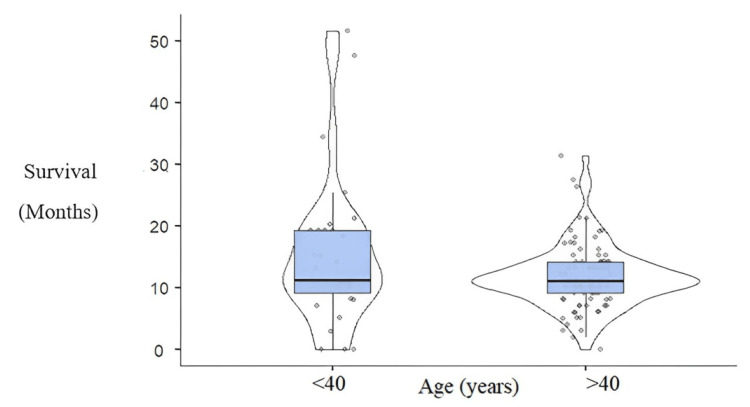
Overall survival in age-related groups (p=0.004) Survival has been represented by the number of months and age in two categories (≤40 years and >40 years). (p <0.05)

Patients' median age at the time of diagnosis was 51.5 years (SD±16.144), of whom 75.4% were older than 40 years. Young patients <40 years had an OS greater than patients >40 years (p = 0.004) (Figure 1).

In our cohort, 75 patients were male (59.5%), while 51 patients were female (40.4%) (male/female sex ratio of 1.47:1) (Table 1). Performance status was defined as good if the KPS was >70 and poor if the KPS was <70. Seventy-seven percent of patients had a KPS at admission >70, and 10.3% had a KPS at the last evaluation >70 (Table 1).

**Figure 2:**
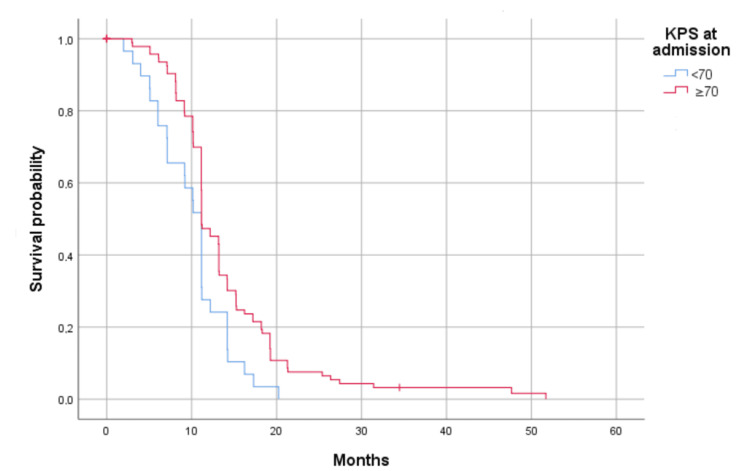
Kaplan-Meier curves showing the significant association of KPS with the overall survival of glioblastoma patients; KPS at admission (p = 0.008). Survival has been represented by probability (percentage) and months. KPS at admission (p = 0.008) (p <0.05) KPS: Karnofsky performance score

**Figure 3:**
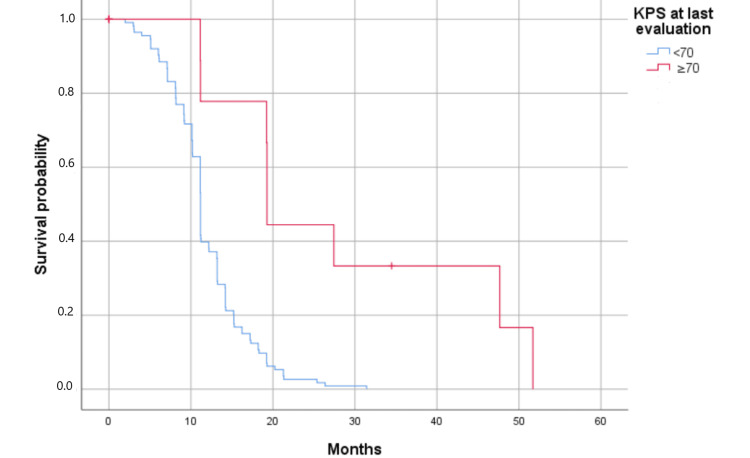
Kaplan-Meier curves showing the significant association of KPS with the overall survival of glioblastoma patients; KPS at last evaluation (p = 0.000). Survival has been represented by probability (percentage) and months. KPS at last evaluation (p = 0.000) (p <0.05) KPS: Karnofsky performance score

Survival analysis showed significant influences of KPS at admission (Figure 2) and KPS at the last evaluation (Figure 3) on OS in univariate analysis (p = 0.008 and p = 0.000, respectively). Furthermore, patients with KPS <70 presented less favorable survival outcomes compared to those with KPS ≥70.

The most common locations were the frontal lobe (44.4%), followed by the temporal lobe (30.2%); 25.4% of patients had parietal and occipital locations (Table 1). All patients had a surgical resection; 50.8% of cases received a subtotal resection; and 49.2% underwent a gross total resection (Table 1).

One hundred and eight received radiation, comprising 85.7% of our sample population, and 94 (74.6% of cases) received chemotherapy with temozolomide according to the Stupp protocol (Table 1). These therapies showed a distinct effect on OS (7.1 months versus 11.2 months), confirming their importance in the management of glioblastoma (p = 0.000).

Survival outcomes after treatment were followed up to the time point when data were retrieved for analysis. The mortality rate at one month after surgery was 0%. The survival rate was 4%. The median OS for patients was found to be 11.17 months (confidence interval: 11.139-11.201). The rate of OS after 12 months of diagnosis was 41.26%. However, after two years of follow-up, the number of survivors was less than 4%.

Immunohistochemical and molecular characteristics

**Figure 4:**
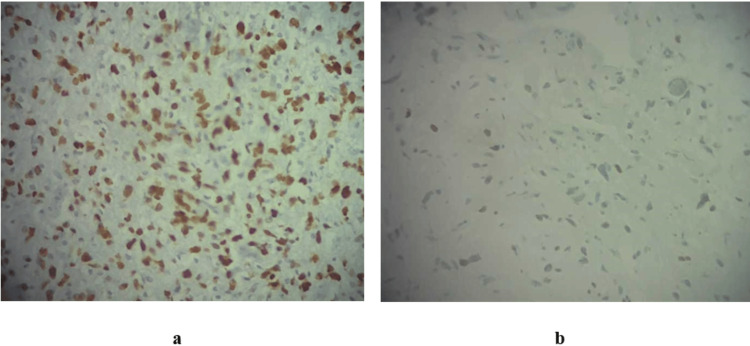
Glioblastoma labeled with two antibodies: p53 (a) and ATRX (b) (x400). These microscopic observations show a nuclear expression of p53 (%) (a) and a loss of ATRX expression in a patient with astrocytoma, grade 4 (b).

An IHC study was conducted using p53 and ATRX antibodies (Figure 4a). P53 immunostaining was >10% in 70 out of 126 patients (55.6%). ATRX expression was lost in 15 out of 126 patients (11.9%) (Figure 4b).

Concerning the IDH-1 gene, the point mutation localized at codon 132 (CGT→CAT) was identified in 13.5% (17/126) of cases.

Clinical correlation of p53 status

**Figure 5:**
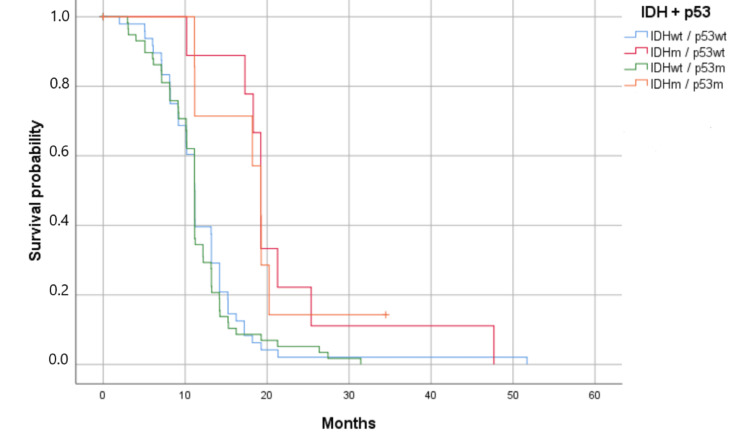
Kaplan-Meier survival estimates by IDH mutation and p53 mutation (p = 0.001). Survival has been represented by probability (percentage) and the number of months. m: mutant; wt: wild-type Patients with IDHm and p53wt had the best overall survival (p < 0.05).

Associations of p53 with clinicopathological parameters were studied in 126 patients. For p53 immunostaining, cases were separated into four categories based on IDH and p53 status (Figure 5). Statistical analyses didn’t show an impact of clinical characteristics on p53 expression (p >0.05). Furthermore, p53 alone had no significant impact on survival (p = 0.726). On the other hand, the association between IDH and p53 statuses showed an impact on patients' OS (p = 0.001). Patients with IDH mutations had the best overall survival (19.230 months) (Figure 5).

Clinical correlation of ATRX status

ATRX status was analyzed in 126 patients; 15 of those cases (11.9%) presented an ATRX mutation. Patients with wild-type ATRX were significantly older (54 years versus 39 years, p = 0.000). The group of patients with the ATRX mutation had a KPS at admission of 90% vs. a KPS of 80% in the ATRX wild-type group (p = 0.278). The ATRX mutation group had a KPS at the last evaluation of 60% vs. a KPS of 40% in the ATRX wild-type group (p = 0.049).

**Figure 6:**
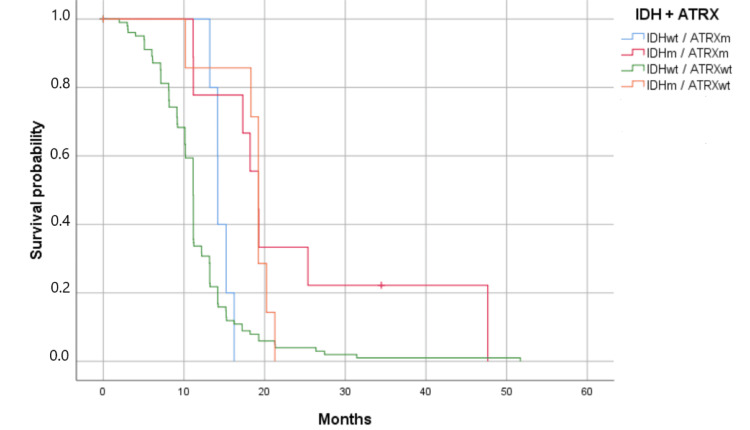
Kaplan-Meier survival estimates by IDH and ATRX mutations (p = 0.001). Survival has been represented by probability (percentage) and the number of months. m: mutant; wt: wildtype Patients with the IDHm and the ATRXm had the best overall survival (p = 0.001).

The median lifespan was 11.17 months for patients with ATRX wild-type, compared with 16.23 months for those with the ATRX mutation (p = 0.045). The association between IDH and ATRX status clearly shows an impact on patients' OS (p = 0.001). Patients with IDH mutations had the best OS (19.233 months) (Figure 6).

Clinical correlation of p53 and ATRX status

**Figure 7:**
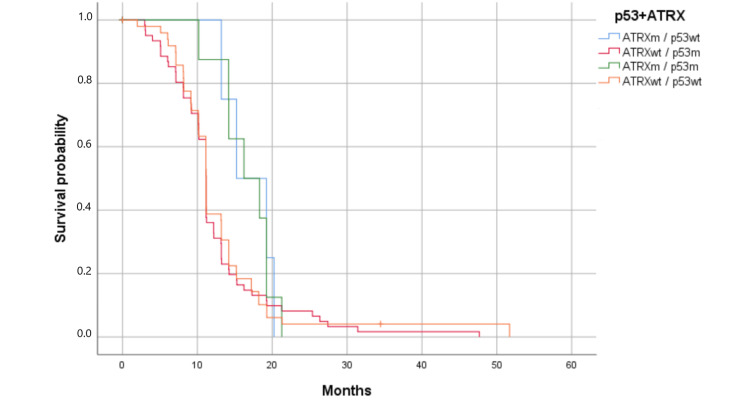
Kaplan-Meier survival estimates by p53 and ATRX mutations (p = 0.242). Survival has been represented by probability (percentage) and the number of months. m: mutant; wt: wildtype Patients with the ATRX mutation and p53 normal had the best survival; the association was not significant (p > 0.05).

Cases were divided into four groups: Group A, ATRX mutant/p53 mutant; Group B, ATRX mutant/p53 wild-type; Group C, ATRX wild-type/p53 mutant; and Group D, ATRX wild-type/p53 wild-type. Kaplan-Meier curves showed that prognosis differed among the four groups. Group B had the best prognosis (OS = 16.23 months) and Group C had the poorest one (OS = 11.17 months) (Figure 7). The association of ATRX loss with p53 overexpression was not as robust in these tumors (p = 0.242).

Clinical correlation of p53, ATRX, and IDH status

The association of IDH, p53, and ATRX status was also studied in 126 patients. The statistical study revealed that the group of patients with IDH mutant/ATRX mutant/p53 wild-type had the best prognosis (OS = 27.393 months), and patients with IDH wild-type/ATRX wild-type/p53 mutant had the poorest one (OS = 11.255 months) (p = 0.015).

## Discussion

Glioblastoma and astrocytoma, grade 4, are the most common primary aggressive brain tumors. The poor prognosis of these tumors is generally due to their ability to recur and resistance to therapies [15]. Recently, advances in molecular testing of gliomas have led to a better understanding of the pathogenesis and biology of these tumors. Classification of glial tumors into different prognostic groups enables us to establish appropriate protocols for better patient management [16]. Several studies have shown the relevance of numerous factors in the diagnosis and prognosis of glioblastoma patients, such as age, KPS and IDH, ATRX, and TP53 status. However, the impact of the latter remains subject to contradiction [15].

In this study, we examined associations between clinicopathological parameters and overall survival in 126 patients with grade 4 glioblastoma/astrocytoma. After follow-up, only 4% of patients were still alive, with other studies suggesting a five-year relative survival of less than 5% [17].

Our results showed that sex, tumor site, and surgical approach did not affect the prognosis. Young age (<40) and KPS at the last evaluation (≥70) were associated with significantly better patient survival (19.27 months vs. 11.17 months, p = 0.000). This result is in line with other studies suggesting that low KPS is an independent risk factor for death in these patients [18].

In addition, we evaluated the association between the expression of IDH, ATRX, and p53 and clinicopathological, radiological, and therapeutic data. We investigated the prognostic value of these biomarkers and their impact on overall survival parameters.

The IDH1R132H mutation has been detected in 13.5% of cases, particularly in young patients, compared to 10% and 12.9% in previous studies [19-21]. In line with several studies, this mutation was associated with a significant increase in OS for patients [17, 22, 23].

P53 nuclear staining ≥10% predicted the TP53 mutation with 84.8% sensitivity and 96.7% specificity [24], [25]. Discrepancies between the two tests could be explained by the presence of TP53 sequence alterations in intronic regions not covered by the primers used for sequencing. In addition, weak or negative immunostaining for p53 could be misinterpreted, particularly in the case of nonsense mutations that do not result in p53 overexpression and therefore will not be detected by immunohistochemistry. The overexpression of p53 was identified in 55.6% (70/126) of patients, compared to 48.4% and 50.8% in the literature [26, 9]. Contrary to some studies that reported that TP53 mutations were associated with reduced median survival, we didn’t observe any association between p53 status and patient survival [26, 27]. Kaplan-Meier analysis showed that patients with p53 overexpression had slightly poorer overall survival than patients without this mutation, even though there was no statistical significance. Our result was in line with previous studies [13, 26].

Ramamoorthy and Smith have shown that in the lack of ATRX, the histone variant macroH2A1.1 binds to tankyrase 1 polymerase, preventing it from localizing on telomeres and resolving cohesion, thus promoting recombination between sister telomeres. Forced resolution of this event induces genomic instability and hinders cell growth [28].

Regarding the detection of ATRX mutation, several studies suggest that gene panel next-generation sequencing analysis (NGS) was less sensitive than IHC in the detection of loss of ATRX expression [29]. This may be explained by epigenetic mechanisms resulting in the loss of ATRX or the presence of mutations in intronic or promoter regions not covered by the designed amplicons [29, 30]. However, the two techniques cannot be compared since NGS shows the mutation at the gene level, whereas IHC shows protein expression. Loss of ATRX was identified in 11.9% of cases, compared to 15.3% in another study [26], and has been significantly associated with better overall survival in high-grade glioma patients. This result is consistent with other studies [8, 9]. Patients with the ATRX mutation were significantly younger (39 years versus 54 years, p = 0.000) and had a KPS of 90% at admission vs. a KPS of 80% in the ATRX wild-type group (p = 0.278). The KPS at the last evaluation was 60% vs. a KPS of 40% in the ATRX wild-type group (p = 0.049).

In their studies, Zhou et al. and Xie et al. showed that the association of ATRX and IDH mutations, particularly in young patients, had a better prognosis and was associated with a high survival rate in grade 4 astrocytoma [8, 9]. Our study also highlighted the prognostic role of the ATRX mutation in a similar group. In our cohort, most patients who were IDHR132H mutants also had a loss of ATRX. However, other patients may have alternative mutations other than R132H, such as R132C and R132G. Thus, it would be interesting to explore other mutations that may affect the IDH gene, although they are in the minority. 

Thus, testing for the ATRX mutation may have utility in the clinical management of high-grade glioma patients. As reported in a previous study [24], we observed that the association of ATRX loss with either TP53 mutation or p53 overexpression was not robust in these tumors (p = 0.242). Despite these findings, several limitations exist in this study, including its retrospective nature. Hence, this investigation is limited to the constraints of such studies. Furthermore, the sample size was small.

## Conclusions

In summary, this study identified genetic alterations in IDH, ATRX, and TP53. In addition, we investigated the relationships between ATRX, p53, and IDH1 expression and clinicopathological parameters in high-grade gliomas.

Based on the results presented, the ATRX and IDH mutations conferred a survival advantage for glioma patients. The clinical management of glioma patients should therefore be targeted according to molecular features. These findings may be a potential therapeutic target for high-grade glial tumors, hence the need for further investigation.
